# Identification of novel blood-based extracellular vesicles biomarker candidates with potential specificity for traumatic brain injury in polytrauma patients

**DOI:** 10.3389/fimmu.2024.1347767

**Published:** 2024-03-12

**Authors:** Cora R. Schindler, Jason A. Hörauf, Birte Weber, Inna Schaible, Ingo Marzi, Dirk Henrich, Liudmila Leppik

**Affiliations:** Department of Trauma-, Hand- and Reconstructive Surgery, University Hospital Frankfurt, Goethe-University, Frankfurt am Main, Germany

**Keywords:** traumatic brain injury, extracellular vesicles, biomarkers, epitopes, polytrauma

## Abstract

**Objective:**

The goal of this study was to identify changes in extracellular vesicles (EV) surface proteins specific to traumatic brain injury (TBI), which could be used as a diagnostic and prognostic tool in polytrauma patients.

**Summary Background Data:**

Known serum TBI-specific biomarkers (S100B, NSE, and GFAP), which can predict the severity and outcome of isolated TBI, lose their predictive value in the presence of additional extracranial injuries. Extracellular vesicles (EVs) are released from cells in response to various stimuli and carry specific cargo/surface molecules that could be used for tracking injury-responding cells.

**Methods:**

EVs were isolated using size exclusion chromatography (SEC) from the plasma of two groups of patients (with isolated TBI, ISS≥16, AIShead≥4, n=10; and polytraumatized patients without TBI ISS≥16, AIShead=0, n=10) collected in the emergency room and 48 h after trauma. EVs’ surface epitope expression was investigated using a neurospecific multiplex flow cytometry assay and compared with healthy controls (n=10). Three enrichments of EV epitopes found to be specific to TBI were validated by western blot.

**Results:**

The expression of 10 EV epitopes differed significantly among the patient and control groups, and five of these epitopes (CD13, CD196, MOG, CD133, and MBP) were TBI-specific. The increased expression of CD196, CD13, and MOG-positive EVs was validated by western blot.

**Conclusion:**

Our data showed that TBI is characterized by a significant increase of CD13, CD196, MOG, CD133, and MBP-positive EVs in patients’ plasma. A high level of MOG-positive EVs negatively correlated with the Glasgow Coma Scale score at admission and could be an indicator of poor neurological status.

## 1 Introduction

Worldwide, severe traumatic brain injury (TBI) and polytrauma remain major causes of death for people younger than 45 years (1). With the improvements in trauma care achieved over the last decades, the all-cause mortality of polytrauma patients admitted to the ICU decreased, whereas TBI-related mortality became a leading cause of death in trauma (2). High brain injury-related mortality and poor outcomes could partially be explained by the trauma mechanism, which can be divided into primary brain injury, direct neuronal damage caused by mechanical forces, and secondary injury, which over the course of several hours or days entails physiological, cellular, and molecular changes, like blood-brain barrier disruption, inflammation, excitotoxicity, mitochondrial dysfunction, and oxidative stress (3). Secondary injury is one of the main reasons for worsening patient outcomes (4) and its prevention is key in acute TBI management. Due to the range of clinical presentations that depend on each individual, type and severity of the injury, gender, and age, the understanding, diagnosis, and treatment of TBI especially in the presence of extracranial injuries are not trivial. Therefore, new clinically reliable neurological markers that could help to assess the severity of injuries at an early stage and determine their pathophysiology are of great interest.

Liquid biopsy biomarkers that can be analyzed soon after injury can offer a very convenient method of diagnosis and prognosis. Hypothesizing that induced by trauma acute axonal, astroglial, and neuronal injury and neuroinflammation could result in the appearance of neurospecific proteins in the bloodstream, several serum protein biomarkers, such as calcium-binding protein B (S100B) (5), neuron-specific enolase two (NSE) (6), gliafibrillary acidic protein (GFAP) (7) or protein combinations (8) were identified. These protein biomarkers already showed promising results in predicting the severity and outcome of isolated TBI (9, 10); however, the predictive value of these markers in the case of polytrauma is significantly lower (11). In addition, the overall use of brain cell-derived proteins as TBI biomarkers should be considered with caution since their concentration in the blood is affected by permeability of the blood-brain barrier and proteolytic degradation (12).

Extracellular vesicles (EVs), a heterogeneous group of cell-derived membrane-enclosed vesicles known to participate in cell-cell communication (13), are currently the subject of intense research as a source of potential biomarkers (14). EVs are secreted by most types of cells in response to various stimuli and carry diverse cargo including nucleic acids, proteins, and lipids implicated in intercellular communication and protected from degradation in the bloodstream (15). Furthermore, EVs carry biologically active surface proteins (derived from the cell of origin) that may be involved in cell-cell communication and post-injury pathology (16) and could be used for tracking injury-responding cells. In polytrauma patients, it was shown that circulating EVs were changed in number, size, and cargo content (17–19) and that cell-specific EVs’ surface proteins reflected the injury pattern (20). All the above, together with the fact that EVs can cross the blood-brain barrier (21, 22), suggests that EVs might be appropriate candidates as blood biomarkers for assessing the biochemical and molecular status of neurological injury in polytrauma patients (20, 23).

In the present study, we hypothesized that TBI-induced damage of brain cells and neuroinflammation could lead to the release of neurospecific EVs into systemic circulation, making TBI-specific biomarkers usable in polytrauma patients. To investigate this, we performed a multiplex comparison of plasma EVs’ surface proteins in two groups of patients (with isolated TBI and polytrauma patients without TBI) and healthy controls in order to identify these TBI-specific EVs populations.

## 2 Materials and methods

### 2.1 Study design

All experiments were performed with ethical approval given by the Local Ethics Committee of the University of Frankfurt (approval ID 89/19) in accordance with the Declaration of Helsinki and following STROBE guidelines (Elm et al., 2008) and with obtained written informed consent for enrolled patients and volunteers. The study includes traumatized patients admitted to the Frankfurt University Hospital Level 1 Trauma Center (Frankfurt am Main, Germany) between, 2016 and, 2020.

The study includes patients with isolated traumatic brain injury (TBI, ISS≥16, AIS_head_≥4, *n=*10), polytrauma patients without TBI (PT, ISS≥16, AIS_head_=0, *n=*10), and healthy volunteers (*n=*10); patients’ demographic and clinical characteristics are presented in **Table 1**. Exclusion criteria were previously known chronic, systemic inflammatory or metabolic syndromes, polyneuropathy, critical illness syndrome, neuro-degenerative diseases (e.g., Dementia and Parkinson’s disease), chronic alcohol abuse, organic brain syndromes (e.g., epilepsy and schizophrenia), stroke, post-traumatic resuscitation, minor age < 18 years, and sepsis. Blood samples were collected at admission in the emergency room and 48 h later and kept on ice, and plasma was gained by 15 min centrifugation at, 3500g (4°C). Prior to EV isolation, plasma was additionally cleared via 30 min centrifugation at, 16000g (4°C). EVs were isolated from 100 µl of plasma by means of size exclusion chromatography (SEC) (EX03-50, Cell guidance system, Cambridge, UK) as described previously (18).

**Table 1:** Demographic and clinical data of patients and healthy controls.

	Healthy *n*=10	TBI *n*=10	Polytrauma *n*=10	
male, *n*=	6*	8	9	* *P ≤* 0.05
Age (years)	40 (37-54)	49 (26-73)	41 (29-54)	*P >*0.05
Accident, (*n*)				
Traffic Fall>3m Fall<3m Violence Others	n/a	4 - 3 2 1	5 4 - 0 1	–
ISS	n/a	26 (25-32)	34(29-40)	P *≤* 0.05
GCS_Pre [pts; median (IQR)]_ GCS_ER_	n/a n/a	6.2 (3-10.75) 4.8 (3-5.5)	15 (13-15) 15 (9.75-15)	*P ≤* 0.05 *P ≤* 0.05
ICU (days; mean ± SD)	n/a	10.5± 9.59	16.70± 12.06	*P ≤* 0.05
ETI (days; mean ± SD)	n/a	5.11± 7.42	3.33± 2.18	*P >*0.05
Outcome (*n=*)				
GOS 5 GOS 4 GOS 3 GOS 2 GOS 1 (dead) Palliative	n/a	1 3 1 1 3 1	4 4 2 0 0 0	–

EV particles’ number and size distribution were determined by nanoparticle tracking analysis (NTA) (Nanosight NS500, Malvern Panalytical, Kassel, Germany). Protein concentration was measured by Coomassie Plus (Bradford) Assay (Thermo Fisher Scientific, Rockford, IL, USA).

### 2.2 EV surface epitopes profiling

The MACSPlex EV Kit Neuro (prototype product, Miltenyi Biotec, Bergisch Gladbach, Germany) was used to quantify EV surface epitopes. This kit comprises two capture bead populations, each coated with different monoclonal antibodies against 37 (panel A) and 32 (panel B) EV surface antigens (the list of bead populations is shown in **Supplementary Table S1**). Isolated EVs (20 µg protein equivalent) from each sample were first incubated with surface epitope-specific antibodies coupled with fluorescent-labeled beads (either Panel A or B) and then with CD9, CD63, and CD81 Exosome Detection Reagent according to the manufacturer’s instructions and analyzed by flow cytometry analysis (BD FACSCanto II, FACS DIVA Software, Heidelberg, Germany). For each sample, the resulting APC-A values were normalized on mean APC-A values of a total amount of EVs measured by CD63, CD81, and CD9, and the group mean values were calculated and compared among the groups.

### 2.3 Western blot analysis

To validate the results of MACSPlex analysis, enrichment of EV epitopes was analyzed on EV samples (20µg protein equivalent) by means of western blot. For all proteins except CD81, the gel separation was performed under reducing conditions. Antibodies against CD13 (Proteintech, 14553, 1:1000), CD196 (Thermofischer, 14-1969-80, 1:1000), MOG (BioLegend, 859901, 1: 1000), CD81 (Invitrogen, 10630D, 1:1000), and either anti-rabbit IgG, horseradish peroxidase (HRP)-linked antibody (Cell signaling Technology, #7074,1:2000), or anti-mouse IgG-HRP-linked antibody (Cell signaling Technology, #7076,1:2000) were used accordingly. Relative signal intensity of CD13, CD196, and MOG was calculated as the ratio of background-subtracted signal intensities [measured with ImageJ software (24)] of the band of interest and either signal intensity of CD81 or total proteins (25).

### 2.4 Statistical analysis

Categorical or continuous variables of clinical and demographic data with skewed distributions were summarized using medians with interquartile ranges (IQR). Categorial data were analyzed by means of a two-sided Fisher’s exact test. For all other comparisons, the non-parametric Kruskal–Wallis test with Bonferroni–Holm corrected Conover–Iman *post hoc* analysis was applied using the statistical software Bias 11.10 (Epsilon-Verlag, Darmstadt, Germany). Spearman’s rank correlation coefficient was calculated to determine mathematical associations between EV epitopes expression and injury characteristics using the Statistical Package for Social Sciences (SPSS for Mac^©^), version 26 (SPSS Inc., Chicago, IL, USA). Results were considered statistically significant when P≤ 0.05.

## 3 Results

### 3.1 Patients clinical characteristics

Overall, 10 isolated TBI (AIS_head_ ≥ 4; ISS ≥16, TBI group) and 10 polytrauma (without TBI) (AIShead = 0; ISS ≥ 16; PT group) patients admitted to the trauma center met the inclusion criteria and were enrolled in the study. The demographic and clinical characteristics of the patients are shown in **Table 1**. The leading trauma mechanisms in both groups were traffic accidents (TBI n=4 and PT n=5) and falls (TBI n=3 and PT n=4). The median Injury Severity Score (ISS) was significantly lower in the TBI patients’ group than in the PT group (26 vs. 34, P = 0.042). Similarly, the pre- and in-hospital Glasgow Coma Scale (GCS) scores were significantly lower (6.2 vs. 15; 4.8 vs. 15; P*<* 0.05) in the TBI group. The outcome of patients with TBI was significantly worse than that of polytrauma patients, thus, n=3 patients died, two patients were in a vegetative state (GOS 2) or severely disabled (GOS 3), and only one of the TBI patients recovered (GOS 5). In comparison, none of the PT patients died, four of these patients were moderately disabled (GOS 4), and four of them recovered (GOS 5). Ten healthy volunteers were recruited representing healthy controls. Compared to the control group, the ages of both patient groups were roughly equivalent, but there were significantly more men.

### 3.2 EVs surface epitope expression

**Figure 1:**
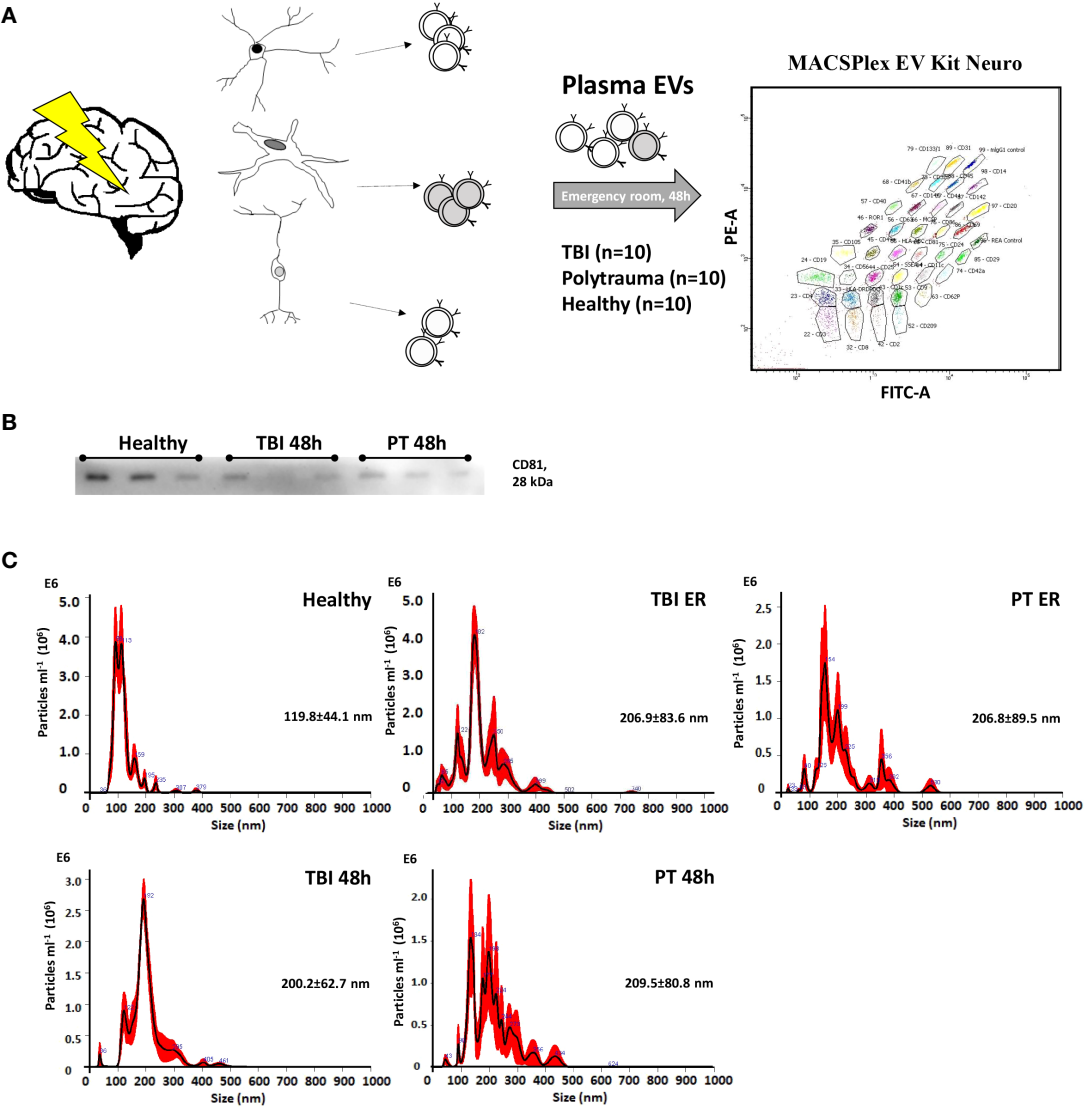
Characterization of plasma-isolated exosomes. **(A)** EVs were isolated from patients’ plasma (Traumatic brain injury, TBI; Polytrauma, PT) and healthy controls and were analyzed with MACSPlex EV Kit Neuro. **(B)** Representative Western-blot analysis of CD81 expression in EVs collected from healthy volunteers, TBI patients (48h time point), and PT patients (48 h time point). **(C)** Representative results of NTA analysis performed with EV isolates (1 to 100 dilution) of healthy volunteers; TBI emergency room, (ER); TBI 48h; PT ER; PT 48h plasma samples. Mean particle size (nm) is provided on the graphs.

**Figure 2:**
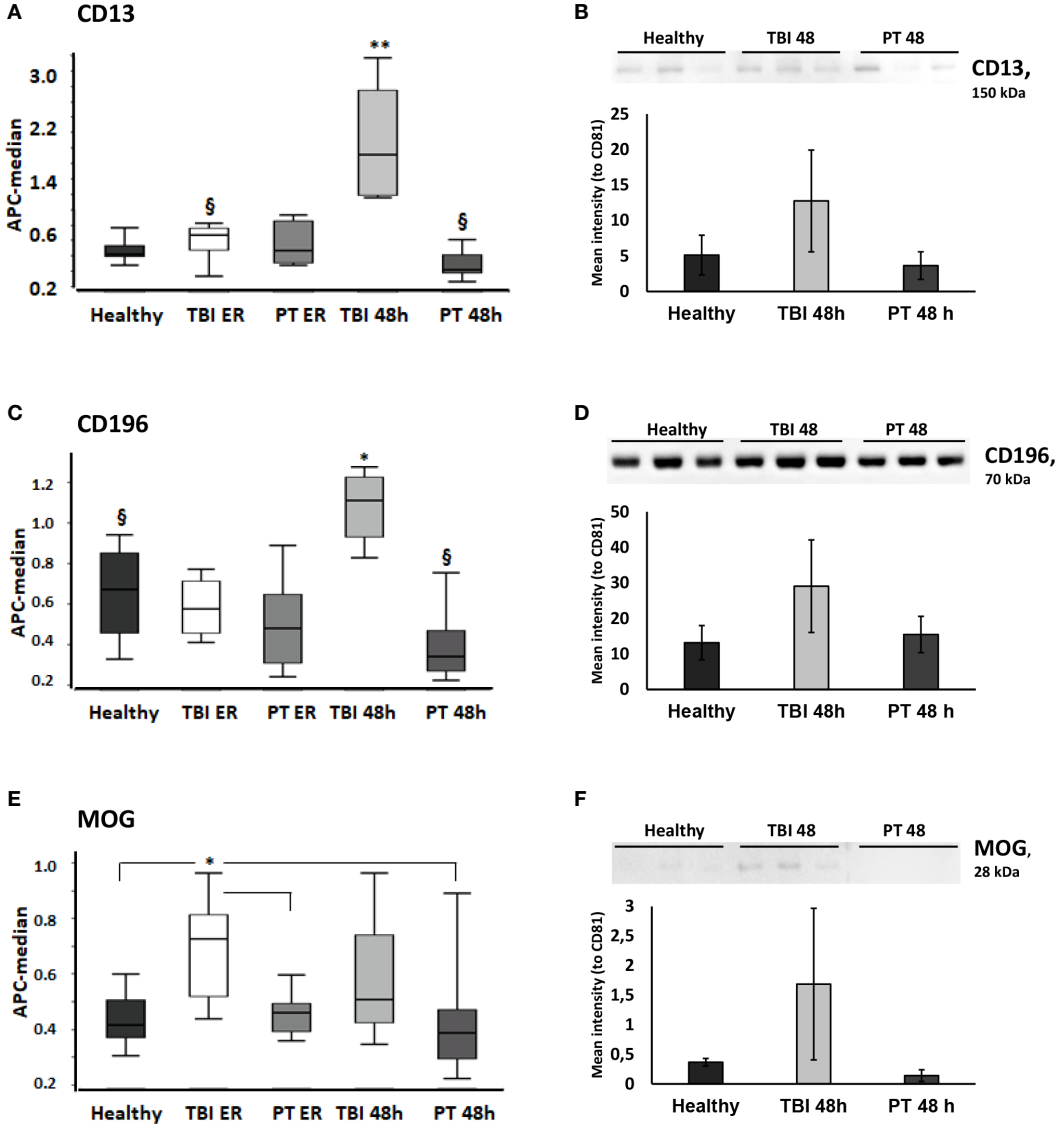
CD13, CD196, and MOG-expressing EVs are significantly increased in the plasma of traumatic brain injury (TBI) patients. CD13+ and CD196+ EVs were increased in TBI patients at 48h and MOG (Myelin Oligodendrocyte Glycoprotein)+ EVs were increased in TBI patients at the ER time point. **(A)**CD9/CD63/CD81 normalized APC median intensity signal for the EV surface protein CD13 differed between TBI patients and PT patients/healthy controls in the MACSPlex analysis. **(B)** Western blot and normalized Western blot quantification for CD13+ EVs. CD13 was further evaluated by Western blot (20 μg protein was loaded in each lane) in plasma EVs from healthy, TBI 48h, and PT 48h patients (n = 3). CD13 was normalized to the CD81 expression of the same samples and the relative signal intensity values are shown in the figure. **(C)** CD196 EV surface expression differed between TBI and PT patients and healthy controls in the MACSPlex analysis. **(D)** The increased expression of CD196+ EVs was confirmed by Western blot analysis. **(E)** MOG+ EVs were significantly increased at the ER time point in the TBI group compared to healthy controls and polytrauma patients (ER and 48h). **(F)** The Western blot analysis and quantification further confirmed the results obtained in MACSPlex analysis. Results are shown as boxplots of the median. * p<0.05; ** p< 0.01; ^§^ p<0.05 among marked groups only.

**Figure 3:**
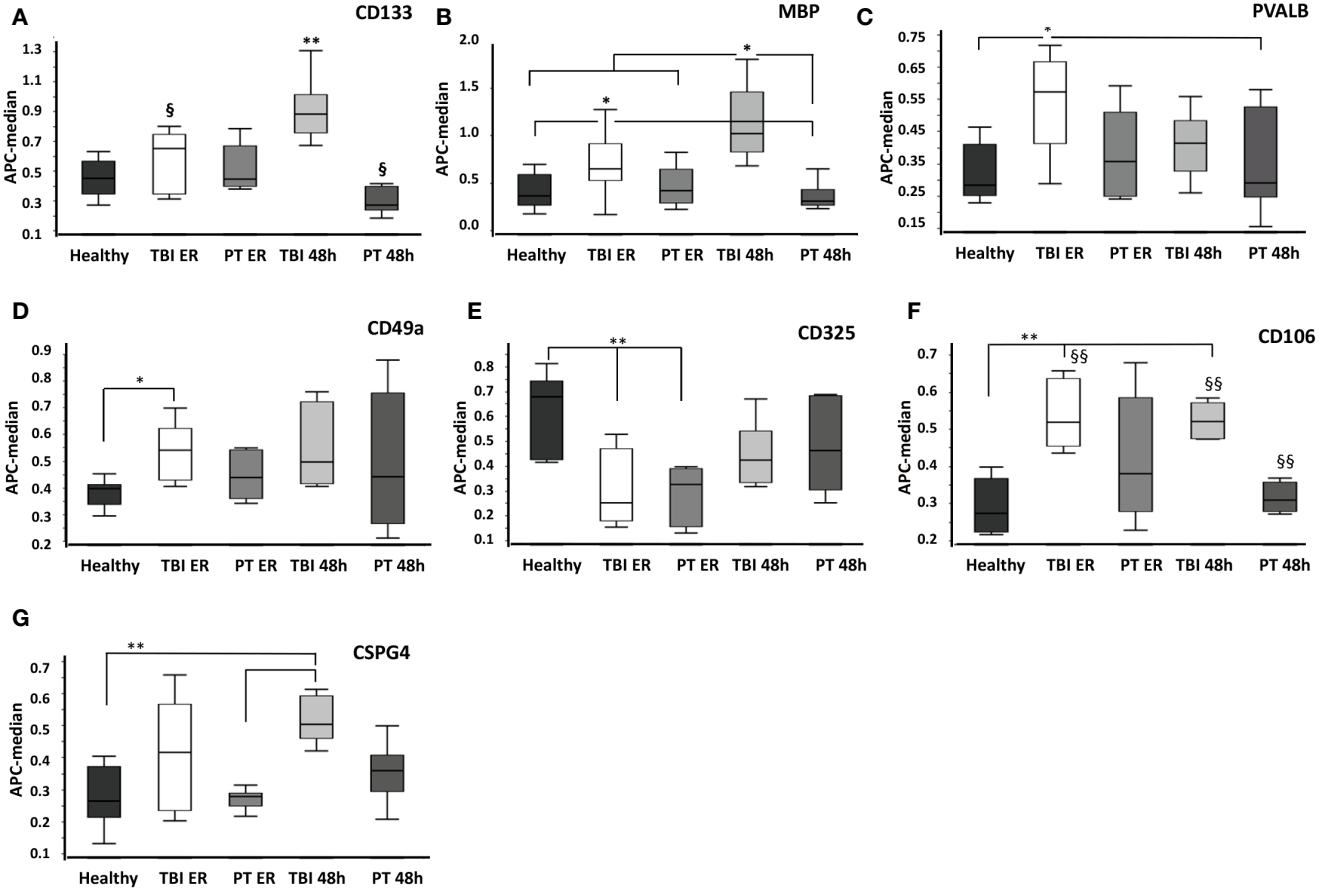
Comparison of surface protein expression in EVs from healthy, traumatic brain injury (TBI), and polytrauma (PT) patients at the emergency room (ER) and 48 h time points. Total EVs were isolated from healthy volunteers’ plasma (healthy, *n*= 10), TBI patients’ plasma collected at the emergency room (TBI ER, *n*= 10) or 48h later (TBI 48h, *n=* 10), and polytrauma patients’ plasma (emergency room time point - PT ER, 48h time point - PT 48h, each *n=* 10). **(A)** CD133+ EVs were significantly increased in TBI patients at 48h compared to all other groups. **(B)** MBP+ EVs were prevalent in TBI patents. The expression of PVALB+ **(C)**, CD49a+ **(D)**, CD325+ **(E)**, CD106+ **(F)**, and CSPG4+ **(G)** EVs was increased in TBI patients; however, it is not TBI-specific. * p<0.05; **<0.01. ^§^ p<0.05; ^§§^ p<0.01 among marked groups only.

In order to compare EV surface epitopes in both groups of patients and healthy controls, EVs were isolated and characterized as a small EV fraction (mean size ≤210 nm). Exosomal marker expression (CD9, CD63, and CD81) was shown by means of MACSPlex analysis, and CD81 expression was further demonstrated with western blot (**Figure 1**). EVs were quantified using protein concentration (**Supplementary Table S2**), and the size distribution of representative samples was accessed with the NTA assay (**Figure 1**). The EV surface epitopes were assessed in both groups of patients and controls with multiplex bead-based flow cytometry assay. Out of all analyzed EV epitopes (listed in **Supplementary Table S1**), we found that the expression of 10 of them differed significantly among the groups and five of these 10 epitopes were TBI-specific (**Figures 2**, **3**). The enrichment of three out of five TBI-specific EV epitopes was additionally validated in representative EV isolates (n=3) by western blot analysis, and two quantification strategies (normalization against exosomal marker CD81 or against total proteins) were applied (**Figures 2B, D, F**, **Supplementary Figure S1**).

In detail, we found that the amount of CD13+ EVs was significantly higher (P<0.05) in TBI patients at the 48 h time point compared to all other groups (**Figure 2A**), and these findings were confirmed with western blot analysis of EV isolates from three representative samples (**Figure 2B**). In addition, the amount of these EVs was significantly lower (P<0.05) in polytrauma patients (48h) compared to TBI patients at the emergency room time point. CD196+ EVs were also found to be significantly increased in TBI patients at 48h after trauma compared to all other groups/time points. We found that the amount of these EVs was significantly decreased in polytrauma patients (48h) compared to controls (**Figures 2C, D**). At the same time, Myelin Oligodendrocyte Glycoprotein (MOG)-positive EVs were found to be significantly increased in the TBI group at the ER time point compared to controls and polytrauma patients (**Figures 2E, F**). The expression of CD133+ and Myelin Basic Protein (MBP) + EVs was also found to be TBI-specific and was significantly increased compared to all other groups in TBI patients at the 48 h time point (CD133) or at both ER and 48 h time points (MBP) (**Figures 3A, B**). The changes in the expression of another five EV epitopes were found to be not TBI-specific. Thus, Parvalbumin (PVALB)+ EV was enriched in the plasma of TBI patients at the ER time point compared to healthy controls and polytrauma patients at 48h (**Figure 3C**). A significant difference in the amount of CD49a+ EVs was found only among healthy controls and TBI patients at the ER time point. CD325+ EV were found to be significantly decreased at ER time point in both groups of patients compared to healthy controls. Enriched expression of CD106+ EV was detected in TBI patients at both time points compared to healthy controls and PT patients at 48h but not in PT patients at ER. In addition, Chondroitin Sulfate Proteoglycan 4 (CSPG4) + EVs were significantly enriched in the TBI 48h group compared to healthy controls and PT ER groups but not in the PT 48 h group.

### 3.3 Correlation between EV surface epitopes expression and clinical parameters

In order to analyze the diagnostic and prognostic potential of the identified differentially expressed EV epitopes, a correlation analysis with clinical parameters was performed. A strong negative correlation (*rho*= -0.829, P=0.042) was found between the concentration of CD13+ EVs, measured at the 48h time point and the endotracheal ventilation time (**Table 2**). For the MOG+ EVs, measured at the emergency room time point, a strong negative correlation was found with the GCS (rho= -0.812, P=0.014). PVALB+ EVs at the ER correlate with the stay-in days in the ICU (rho= 0.783, P=0.013).

**Table 2:** Correlation analysis of EVs’ markers and clinical parameters.

EV-Marker time point	Spearman correlation	GCS	ICU (days)	ETI (days)
CD13 48h	*rho*	0.516	-0.145	**-0.829**
	*P*-value	0.295	0.784	**0.042**
CD133 Emergency Room	*rho*	-0.621	-0.599	0.109
	*P*-value	0.100	0.117	0.816
CD196 48h	*rho*	0.334	-0.058	0.029
	*P*-value	0.518	0.913	0.957
MOG Emergency Room	*rho*	**-0.812**	-0.143	0.054
	*P*-value	**0.014**	0.736	0.908
MOG 48h	*rho*	0.203	-0.319	-0.551
	*P*-value	0.700	0.538	0.257
PVALB Emergency Room	*rho*	0.287	**0.783**	0.699
	*P*-value	0.454	**0.013**	0.054
GOS	*rho*	**0.676**	0.510	-0.130
	*P*-value	**0.046**	0.160	0.738

Bold letters: rho with p value<0.05.

The correlation analysis of the clinical data revealed a significant positive relationship (rho= 0.676, P= 0.046) between the patient’s GCS on admission and the outcome score (GOS).

## 4 Discussion

The clinical presentation of TBI frequently deviates from the radiological findings and has high variability on the individual level, which greatly complicates the planning of therapy and prediction of the patient outcome. Since EV content including cargo and surface proteins can reflect the cell source and cells’ activity, analysis of EV surface signatures in the plasma of trauma patients has the potential to reveal characteristic changes caused by TBI. We compared surface epitope composition in EVs that were isolated from the plasma of healthy controls and two groups of trauma patients—with isolated TBI and polytrauma (without traumatic brain injury).

The patients included in our study have the typical demographic and clinical characteristics of a trauma study group (26): significantly more men with an average age of 49 and 41 years (TBI and PT groups, respectively) were involved in traffic accidents and falls. The ISS and both GCS scores, as expected, were significantly lower in patients with isolated TBI than in the PT group due to monotrauma. Although polytrauma patients had a longer ICU stay, the outcome at discharge was significantly better in this group. Correlation analysis of our clinical demographic data showed a significant correlation between patients’ GCS on admission and outcome score (GOS), which is in accordance with previously published findings in TBI patients (27). This highlights once again how crucial traumatic brain injury is for the overall outcome of polytrauma patients and the urgent need for new diagnostic and prognostic tools.

EV surface profile analysis was performed with the prototype product of Miltenyi Biotec MACSPlex EV Kit Neuro, which is based on well-established MACSPlex technology (28) and includes two new panels of exosome capture beads, each bead coated with antibodies against neuro-associated proteins. We analyzed the expression of 69 different EV-surface proteins and found out that 10 proteins (CD13, CD196, MOG, CD133, MBP, PVALB, CD49a, CD325, CD106, and CSPG4; **Supplementary Table S3**) were differentially expressed, whereas the majority of the proteins were expressed at a similar level in all groups. Importantly, the expression of CD13, CD196, MOG, CD133, and MBP-positive EVs was found to be TBI-specific. Among them, only MOG+EVs were found to be up-regulated at the ER time point, whereas MBP+ EVs were elevated at both time points and the other three EV types were enriched at the 48 h (secondary injury) time point.

Since its discovery, serum/cerebrospinal fluid MBP or Myelin Basic Protein has been regarded as a marker of brain tissue injury in TBI, cerebrovascular accidents, multiple sclerosis (MS), intracranial tumors, and CNS infections (29); however, its expression was also found in the peripheral nervous system (30) which limits the TBI diagnostic potential of this protein in polytrauma patients. MOG (or Myelin Oligodendrocyte Glycoprotein) is an important marker for oligodendrocyte maturation and is one of the best-studied autoantigens for experimental autoimmune models for MS (31). In contrast to MBP, MOG is expressed solely in oligodendrocytes (32). MBP+ and MOG+ oligodendrocyte-derived EVs were suggested as a differential marker of MS progression (33). Oligodendrocyte production of MBP+ EVs was shown to be regulated by cytosolic calcium levels (34). We found a strong negative correlation (*rho*= -0.812) between MOG+ EVs measured in plasma at the emergency room time point and the GCS. In other words, the higher the expression of MOG + EVs at the time of admission, the lower the GCS, and the patient’s neurological status at the time of admission was worse. This suggests that the expression level of MOG+ EVs also reflects the individual clinical injury severity of the patient. Oligodendrocytes are known to be dynamic cell populations, which could proliferate, migrate, and differentiate in response to the injury (35); therefore, it is not clear whether the increased amount of oligodendrocyte-derived EVs in TBI patients reflects tissue damage only or also regenerative activity. The observation that MOG+ EVs were up-regulated at an earlier time point and MBP+EV later shows that these EVs could play different roles during injury course and should be investigated in more detail in the future.

Three other EV populations (CD13+, CD133+, and CD196+) were all found to be elevated at a 48h time point suggesting their involvement in secondary injury and neuroinflammation (36). CD13 protein or aminopeptidase N appeared to be an important epitope of microglia-derived exosomes (MDEs) and the role of these exosomes in neuronal metabolic support and neuropeptide catabolism was suggested (37). We found a strong negative correlation between significantly increased expression of CD13+ EVs and the endotracheal ventilation time of the TBI patients at the 48 h time point, which suggests the possible involvement of these EVs in immune protective reaction. CD133 (Prominin-1) stem- and progenitor cell-marker is known to be expressed also on neural stem cells and their exosomes (neural stem cells derived exosome, NDEs) (38). A recent study reported that NDEs selectively target microglia, function as a microglial morphogen (39), and modulate microglial activation during brain injury (40). It seems that crosstalk between MDEs and NDEs could mediate inflammatory injury but also exert neuroprotective effects, and there is an intercellular feedback loop (40). Interestingly, the expression profiles that we obtained for the CD13+ and CD133+ EVs are quite similar, which could support the suggested cross-talk of these EVs during TBI.

CD196 chemokine receptor 6 or CCR6 is expressed on several types of immune cells, has only one ligand, CCL20 (41), and controls cell migration and immune induction during inflammatory and immunological responses (42). In CNS, CCR6 plays a role in the chemotaxis of pro-inflammatory immune cells to the inflamed sites in the brain (43). Less is known about the role of CD196/CCR6+ exosomes in traumatic injury, and only a few studies describe such particles in different types of cancer (44). Our data show that the amount of CD196+ EVs is increased in TBI patients’ plasma at the 48 h late-injury time point, but the mechanistic explanation of this observation remains to be found.

Another five EVs’ epitopes (PVALB, CD49a CD325, CD106, and CSPG4), found to be differentially expressed in control and patient groups, seem to be rather trauma- and time-point-specific than TBI- specific. This may be explained by the fact that these proteins are expressed and play a role not only in CNS but also in other tissues and, therefore, these EVs could have multiple cell-origins in plasma. Thus, PVALB was shown to have broad expressions in brain and muscle tissues (45). We found that the expression level of PVALB+ EVs at the time of admission correlates with the stay-in days in the ICU (*rho=* 0.783) in TBI patients. This means that a higher level of PVALB+ EVs could be an indicator of clinical injury severity, but the TBI specificity of this marker needs to be verified. CD49a (also known as Integrin Alpha-1) is a dual laminin/collagen receptor expressed in neural and hematopoietic cells and has been described as a marker of tissue-resident memory T cells (46). CD325 (or Cadherin-2) was originally named neural cadherin for its important role in CNS. However, it is also expressed in multiple tissues where it functions as a mediator of cell–cell adhesion (47). CSPG4 (Chondroitin Sulfate Proteoglycan 4) or Neuron-Glial Antigen 2 was originally thought to play a role in regulating the blood-brain barrier and tissue homeostasis but recently was found to function in various cell types and to be upregulated in many aggressive cancers as well (48). It seems to be plausible that EVs expressing these epitopes (PVALB+, CD49a+, CD325+, CD106+, and CSPG4+), in the case of TBI, originates from damaged brain tissues, whereas in polytrauma, the injury to other tissues could induce production of these EVs as well, which decrease the specificity of these potential markers.

This study has several technical limitations. Due to the small number of patients with isolated TBI who matched the study inclusion criteria, only a small number of patients (*n*=10 pro group) were enrolled. For EV isolation, out of the multiple isolation techniques, we chose size exclusion chromatography (SEC) as from experience this method provides sufficient quantity and quality of EVs when working with a low amount of plasma material (17, 20). However, it is known that SEC isolates of EVs could have contaminants of plasma proteins. In MACSPlex analysis, this possible contamination is not expected to have major effects on the results, as quantification includes normalization for exosomal markers (CD9, CD63, and CD81). Nevertheless, in validation experiments with Western blot analysis, we included two normalization protocols—by means of exosome-specific CD81 protein expression and by total proteins. Whereas, results obtained in both ways showed a similar tendency and overall confirmed the MACSPlex findings, the difference among the groups was stronger in the case of normalization for EV-specific protein (CD81). This can be explained by the presence of plasma protein contaminants in SEC EV preparations.

Summing up, our results demonstrated that surface epitope profiles of plasma EVs in patients with isolated TBI and severely injured patients (without TBI) differ significantly. The use of neuro-specific multiplex assay allowed us to identify new potential TBI biomarkers and point out the complexity of the interaction networks between various extracellular vesicles in brain injury. Future research may support the use of these markers in conjunction with well-established diagnostic instruments (such as CT scans and/or intracranial pressure measurements) to determine the degree and severity of brain injury, predict the clinical course and neurological outcome, and help determine the best course of patient treatment (such as surgery versus a wait-and-see approach). In addition, these biomarkers may help to better understand the complex and dynamic pathophysiology of severe traumatic brain damage, particularly secondary brain injury, and suggest potential therapeutic targets for the treatment of TBI.

## 5 Conclusion

Our study revealed a significant increase in CD13+, CD196+, MOG+, CD133+, and MBP+ EVs in the plasma of TBI patients compared to polytrauma patients and healthy controls. A level of MOG-positive EVs at the time of admission shows a significant negative correlation with patients on the Glasgow Coma Scale and could be an indicator of poor neurological status.

## Data availability statement

The raw data supporting the conclusions of this article will be made available by the authors, without undue reservation.

## Ethics statement

The studies involving humans were approved by Local Ethics Committee of the University of Frankfurt (approval ID 89/19). The studies were conducted in accordance with the local legislation and institutional requirements. The participants provided their written informed consent to participate in this study.

## Author contributions

CS: Conceptualization, Data curation, Formal analysis, Funding acquisition, Validation, Writing – original draft, Writing – review & editing. JH: Formal analysis, Validation, Writing – original draft, Writing – review & editing. BW: Data curation, Methodology, Validation, Writing – review & editing. IS: Formal analysis, Investigation, Validation, Writing – review & editing. IM: Conceptualization, Funding acquisition, Resources, Supervision, Writing – review & editing. DH: Conceptualization, Data curation, Resources, Visualization, Writing – review & editing. LL: Conceptualization, Data curation, Formal analysis, Investigation, Methodology, Validation, Visualization, Writing – original draft, Writing – review & editing.

## Supplementary material

Supplementary Figure 1 Western blot quantification for CD13, CD196, and MOG EV expression. CD13, CD196, and MOG were further evaluated by western blot (20 μg protein was loaded in each lane) in plasma EVs from healthy, TBI 48h, and PT 48h patients (*n=* 3). The signal intensity was normalized to the total protein of the same samples and the relative signal intensity values are shown in the figure.
