# Supplementary material for: Identification of novel blood-based extracellular vesicles biomarker candidates with potential specificity for traumatic brain injury in polytrauma patients
Source: Front Immunol. 2024 Mar 12;15:1347767. doi: 10.3389/fimmu.2024.1347767 (PMC10963595; doi:10.3389/fimmu.2024.1347767)
Supplement: Supplementary Figure 1 — Western blot quantification for CD13, CD196, and MOG EV expression. CD13, CD196, and MOG were further evaluated by western blot (20 μg protein was loaded in each lane) in plasma EVs from healthy, TBI 48h, and PT 48h patients (n= 3). The signal intensity was normalized to the total protein of the same samples and the relative signal intensity values are shown in the figure. [file Image_1.pdf]

Supplementary Material

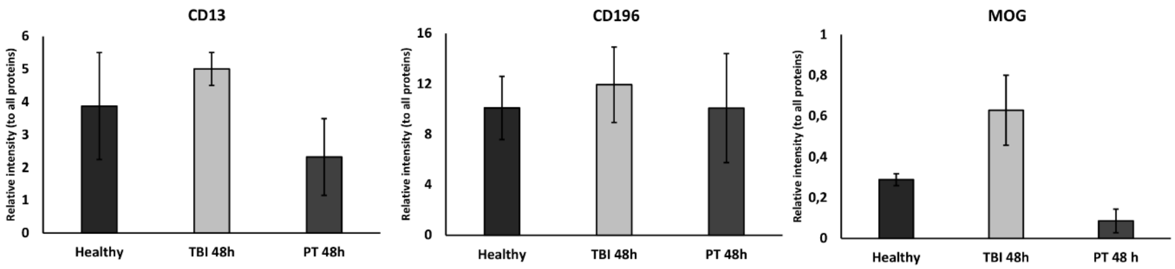

Supplementary figure S1. Western blot quantification for CD13, CD196 and MOG EV expression. CD13, CD196 and MOG were further evaluated by western blot (20 µg protein was loaded in each lane) in plasma EVs from healthy, TBI 48h and PT 48h patients (*n*= 3). The signal intencity was normalized to the total protein of the same samples and the relative signal intensity values are shown in the figure.
