# Supplementary material for: Identification of novel blood-based extracellular vesicles biomarker candidates with potential specificity for traumatic brain injury in polytrauma patients
Source: Front Immunol. 2024 Mar 12;15:1347767. doi: 10.3389/fimmu.2024.1347767 (PMC10963595; doi:10.3389/fimmu.2024.1347767)
Supplement: Supplementary file 2 [file Table_1.docx]

Supplementary Material

**Supplementary Table S1. MACSPlex EV Kit Neuro beads populations.**

|  | Antibody | Bead No. | Name | Isotyp |
| --- | --- | --- | --- | --- |
| Panel A | CD68 | 22 | CD68 | REA |
| Panel A | CD340 | 23 | CD340 | mIgG1k |
| Panel A | CD49a | 24 | CD49a | REA |
| Panel A | CD140a | 32 | CD140a | REA |
| Panel A | CD171 | 33 | CD171 | REA |
| Panel A | CD56 | 34 | CD56 | REA |
| Panel A | CD13 | 35 | CD13 | REA |
| Panel A | BDNF | 42 | aBDNF | REA |
| Panel A | CD31 | 43 | CD31 | REA |
| Panel A | GFAP | 44 | aGFAP | REA |
| Panel A | CD222 | 45 | CD222(aIGF2R) | REA |
| Panel A | CD11b | 46 | CD11b | ratIgG2bk |
| Panel A | CD24 | 52 | CD24 | REA |
| Panel A | CD45 | 53 | CD45 | REA |
| Panel A | CD133 | 54 | CD133/1 | REA |
| Panel A | O4 | 55 | aO4 | mIgM |
| Panel A | Glast | 56 | anti Glast | mIgG2ak |
| Panel A | CD90 | 57 | CD90 | REA |
| Panel A | CX3CR1 | 63 | aCX3CR1 | REA |
| Panel A | EGF | 64 | aEGF | REA |
| Panel A | CD325 | 65 | CD325 | mIgG1k |
| Panel A | Podoplanin | 66 | aPodoplanin | REA |
| Panel A | A2b5 | 67 | aA2B5 | mIgMk |
| Panel A | CD29 | 68 | CD29 | REA |
| Panel A | CSPG4 | 74 | CSPG4(AN2) | ratIgG1 |
| Panel A | CD9 | 75 | CD9 | mIgG1 |
| Panel A | CD63 | 76 | CD63 | mIgG1k |
| Panel A | CD81 | 77 | CD81 | REA |
| Panel A | CD47 | 78 | CD47,h | REA |
| Panel A | PSA-Ncam | 79 | PSA-Ncam | mIgM |
| Panel A | CD44 | 85 | CD44,h | mIgG1k |
| Panel A | CD38 | 86 | CD38,h | REA |
| Panel A | CD49f | 87 | CD49f,h | REA |
| Panel A | CD106 | 88 | CD106,h | REA |
| Panel A | CD119 | 89 | CD119,h | REA |
| Panel A | CD49e | 96 | CD49e,h | REA |
| Panel A | CD54 | 97 | CD54,h | REA |
| Panel A | CD36 | 98 | CD36,h | REA |
| Panel A | CD45RB | 99 | CD45RB,h | REA |
| Panel B | CD18 | 22 | CD18,h | REA |
| Panel B | CD64 | 23 | CD64,h | REA |
| Panel B | CD107a | 24 | CD107a,h | mIgG1k |
| Panel B | CD180 | 32 | CD180,h | REA |
| Panel B | CD196 | 33 | CD196,h | REA |
| Panel B | ADAM17 | 34 | ADAM17 | mIgG2bkappa |
| Panel B | APP | 35 | APP | mIgG1kappa |
| Panel B | CLND | 42 | CLDN5 | mIgG2bkappa |
| Panel B | ENO2 | 43 | ENO2 | mIgG2b |
| Panel B | GALC | 44 | GALC | mIgG2akappa |
| Panel B | GPNMB | 45 | GPNMB | mIgG1 |
| Panel B | MOG | 46 | MOG | mIgG1 |
| Panel B | PLP1 | 52 | PLP1 | mIgG2a |
| Panel B | SLC16A1 | 53 | SLC16A1 | mIgG2b |
| Panel B | NeuN | 54 | NeuN | REA |
| Panel B | Synypsin | 55 | Synapsin1 | REA |
| Panel B | Tau | 56 | Tau | REA |
| Panel B | PLP | 57 | Proteolipid Protein | REA |
| Panel B | MBP | 63 | Myelin Basic Protein | REA |
| Panel B | VGlut2 | 64 | Vesikular-glutamtate-transporter 2 | REA |
| Panel B | GD-2 | 78 | Ganglioside G2 | Fab |
