# Supplementary material for: Identification of novel blood-based extracellular vesicles biomarker candidates with potential specificity for traumatic brain injury in polytrauma patients
Source: Front Immunol. 2024 Mar 12;15:1347767. doi: 10.3389/fimmu.2024.1347767 (PMC10963595; doi:10.3389/fimmu.2024.1347767)
Supplement: Supplementary file 3 [file Table_2.docx]

Supplementary Material

**Supplementary Table S2.**

|  | EV Protein (ng/µl) | | | | |
| --- | --- | --- | --- | --- | --- |
|  | Healthy | TBI ER | PT ER | TBI 48h | PT 48h |
| 1 | 622.86 | 660.29 | 728.00 | 578.57 | 911.57 |
| 2 | 696.29 | 663.86 | 632.29 | 720.71 | 867.86 |
| 3 | 714.71 | 779.86 | 646.00 | 659.43 | 722.71 |
| 4 | 701.57 | 505.00 | 723.86 | 495.43 | 737.00 |
| 5 | 788.86 | 745.57 | 943.57 | 872.57 | 660.43 |
| 6 | 870.57 | 795.00 | 1225.14 | 692.86 | 810.43 |
| 7 | 673.57 | 878.43 | 565.00 | 758.43 | 817.00 |
| 8 | 738.86 | 600.00 | 629.57 | 753.86 | 560.14 |
| 9 | 732.14 | 626.43 | 823.00 | - | 640.43 |
| 10 | 1468.29 | 768.14 | 587.00 | - | 497.00 |

*TBI – traumatic brain injury; PT – polytrauma; ER-emergency room*
