# Supplementary material for: Identification of novel blood-based extracellular vesicles biomarker candidates with potential specificity for traumatic brain injury in polytrauma patients
Source: Front Immunol. 2024 Mar 12;15:1347767. doi: 10.3389/fimmu.2024.1347767 (PMC10963595; doi:10.3389/fimmu.2024.1347767)
Supplement: Supplementary file 4 [file Table_3.docx]

Supplementary Material

**Supplementary Table S3. EV surface proteins.**

| **EV surface protein** | **Also known as:** |
| --- | --- |
| CD13 | ANPEP, Aminopeptidase N |
| CD133 | PROM1, Prominin-1 |
| CD196 | CCR-6, Chemokine (C-C) Receptor 6 |
| CD49a | ITGA1, Integrin Alpha-1 |
| CD325 | CDH2, Cadherin-2 |
| CD106 | VCAM1, Vascular Cell Adhesion Protein 1 |
| MOG | Myelin Oligodendrocyte Glycoprotein |
| MBP | Myelin Basic Protein |
| CSPG4 | Chondroitin Sulfate Proteoglycan 4 |
| PVALB | Parvalbumin |
